# Clinical applications and cadaveric study of the free descending genicular artery perforator flap without the saphenous vein

**DOI:** 10.1186/s12893-024-02481-5

**Published:** 2024-06-15

**Authors:** Xiaolong Zhang, Junyu Chen, Lebin Zhuang, Lingfei Ouyang, Weichao Gui, Zilong Yao, Bowei Wang, Ping Zhang, Bin Yu, Hua Liao, Jijie Hu

**Affiliations:** 1 Division of Orthopaedics and Traumatology, Department of Orthopaedics, Nanfang Hospital, Southern Medical University, Guangzhou, China; 2 Baiyun Branch, Southern Hospital of Southern Medical University, Guangzhou, 510420 China; 3 Department of Human Anatomy, School of Basic Medical Sciences, Southern Medical University, Guangzhou, Guangdong China

**Keywords:** Reconstructive surgery, Perforator flap, Descending genicular artery, Saphenous vein

## Abstract

**Background:**

The descending genicular artery (DGA) and medial thigh region have been underused as donor sites for perforator flaps. This study evaluated the anatomical relationship between the perforators of the DGA and the saphenous vein (SV) to review the clinical applications of the free descending genicular artery perforator (DGAP) flap for locoregional reconstruction.

**Methods:**

Fifteen cadavers were arterially perfused with red latex and dissected. Thirty-one patients with extremity tissue defects were treated with a free DGAP flap, including six patients who received a chimeric flap. The minimum distance between the DGAP and the SV was measured during surgery.

**Results:**

In all patients, the skin branch of the descending genicular artery was found in the medial femoral condyle plane in front of the SV. The average distance between the descending genicular artery perforator and the SV was 3.71 ± 0.38 cm (range: 2.9–4.3 cm). Thirty flaps survived completely, and one flap developed partial necrosis; however, this flap healed two weeks after skin grafting. The average follow-up time was 11.23 months.

**Conclusions:**

We conclude that the SV can be preserved when harvesting the descending genicular artery perforator flap, causing less damage to the donor site and having no effect on flap survival. The free descending genicular artery perforator flap without the SV is a better therapy for complicated tissue defects.

**Supplementary Information:**

The online version contains supplementary material available at 10.1186/s12893-024-02481-5.

## Background

The medial knee has commonly been used as a donor site for free skin flaps [1]. The free saphenous flap from the medial knee area, using the saphenous artery (SA) arising from the descending genicular artery (DGA), was first described by Acland in 1981 [2]. However, harvesting the SV and saphenous nerve during this procedure can interrupt distal venous return and affect the sensation of the skin on the lower leg and medial foot [3]. There is also a risk of lower limb swelling, numbness, and rash on the medial skin. Even with the use of digitally assisted flap designs in recent years, controlling injury to the donor site with SA flaps remains challenging [4]. Karamürsel and Celebioğlu [5] introduced the clinical application of a direct cutaneous perforator flap of the DGA, which offers protection to the saphenous nerve and causes less damage compared to the SA flap. Furthermore, chimeric flaps related to the DGA have been developed in recent years for the treatment of limb tissue defects, bone nonunions, and tendon reconstruction, showing satisfactory results [6–9].

The name of the saphenous vein is derived from the Greek word ‘safaina’, which does not refer to a ‘shallow vein’ or ‘useless vein’ that can be sacrificed during flap surgery [10]. Except in the ankle, the SV is located on the surface of the deep fascia and is deeper than other superficial veins. Similar to deep veins, the surface of the SV has a dense envelope that separates it from other superficial veins [11]. Therefore, the superficial branch of the SV is often mistaken for its trunk on vascular ultrasound. It is crucial to preserve the SV trunk during the harvesting of the DGAP flap. However, existing studies [12–14] on the anatomical aspects of the DGA have primarily focused on the variations of its branches and the parameters of the branch arteries. These studies do not provide clear answers regarding the relationship between the SV and the DGAP flap. Furthermore, there are no reports mentioning whether the SV should be preserved during the harvesting of the DGAP flap.

We designed the DGAP flap without the SV and saphenous nerve, conducted a cadaveric perfusion study, and treated extremity tissue defects using the DGAP flap. The objectives of this study were to: (1) summarize the anatomical relationship between the DGAP flap and the SV; (2) control injury to the donor site; and (3) summarize the clinical significance and complications of the DGAP flap.

## Materials and methods

### Anatomical dissection

**Fig. 1:**
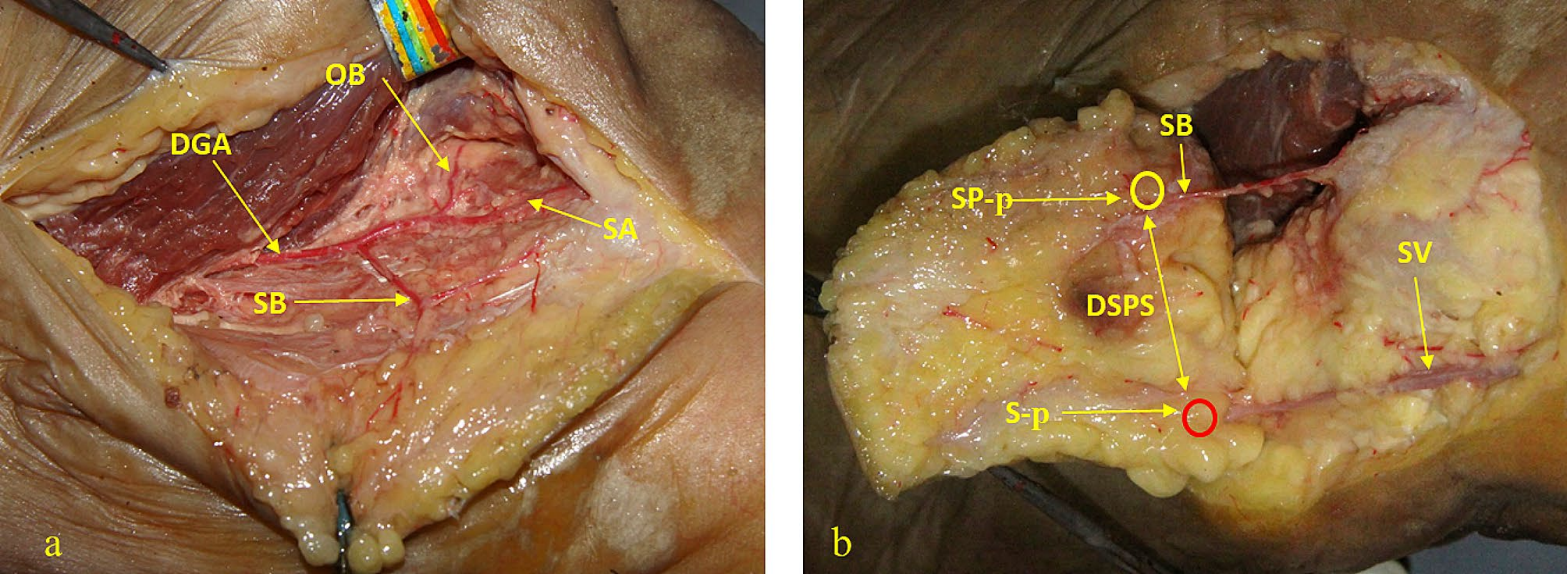
Cadaveric dissection of the medial knee showing the main arteries (**1a**). In this specimen, the SA originates from the distal DGA. The DSPS was measured in each specimen (**1b**). DGA, descending genicular artery; SB, skin branch; OB, osteoarticular branch; SA, saphenous artery; SV, saphenous vein; SP-p, perforator point of the skin branch (yellow circle); S-p, intersection point obtained by making a vertical line from SP-p to the SV (red circle); DSPS, distance between SP-p and S-p

Fifteen fresh human lower-extremity specimens were first intubated via the femoral artery, and blood vessels were washed with 10% formaldehyde (2000–3000 ml) until venous vessels no longer returned blood. After 24 h, the femoral artery was perfused with a red latex mixture at room temperature (24 °C) using a syringe until the toes were flushed red [15]. One centimeter from the medial patella of the specimen, an arc incision was made along the medial midline of the thigh to the proximal end. The skin branch (SB) of the DGA was positioned approximately 4 cm above the knee joint, and its skin entry point was marked as the P point (SP-p). Reverse separation along the SB with protection showed the branches of the DGA. The adductor canal was opened along the DGA. The osteoarticular branch (OB) was found on the anterior and inferior sides of the femoral medial condyle, and the branch artery of the femoral medial muscle was found near the proximal OB (Fig. 1a). The main trunk of the SV was exposed by separating the tissue in the superficial layer of the deep fascia, and the DGAP penetrated the skin anterior to the SV. The intersection point was marked as the S point (S-p), which was obtained by making a vertical line from point P to the SV. The distance between the SP-p and the S-p was designated as DSPS (Fig. 1b). The DSPS of each specimen was measured and recorded.

### Patients

This is a retrospective case series study using a free descending genicular artery perforator flap without the SV. The study was conducted from June 2011 to October 2018 and included 31 patients (24 males and 7 females) with an average age of 30.9 years (range: 4–63 years). Of these patients, 13 had hand or forearm tissue defects, 17 had foot or ankle tissue defects, and 1 case involved early femoral head necrosis after internal fixation of a femoral neck fracture. Six patients were treated with a chimeric DGAP flap, while the remaining patients received a simple DGAP flap. The study collected patient demographics, procedural information, and postoperative details.

This study was conducted in accordance with the principles of the Declaration of Helsinki. All surgeries were performed by orthopedic surgeons from the same group at Nanfang Hospital. All surgical methods were approved by the ethics committee of Nanfang Hospital, Southern Medical University, and all patients provided written informed consent.

### Flap design

**Fig. 2:**
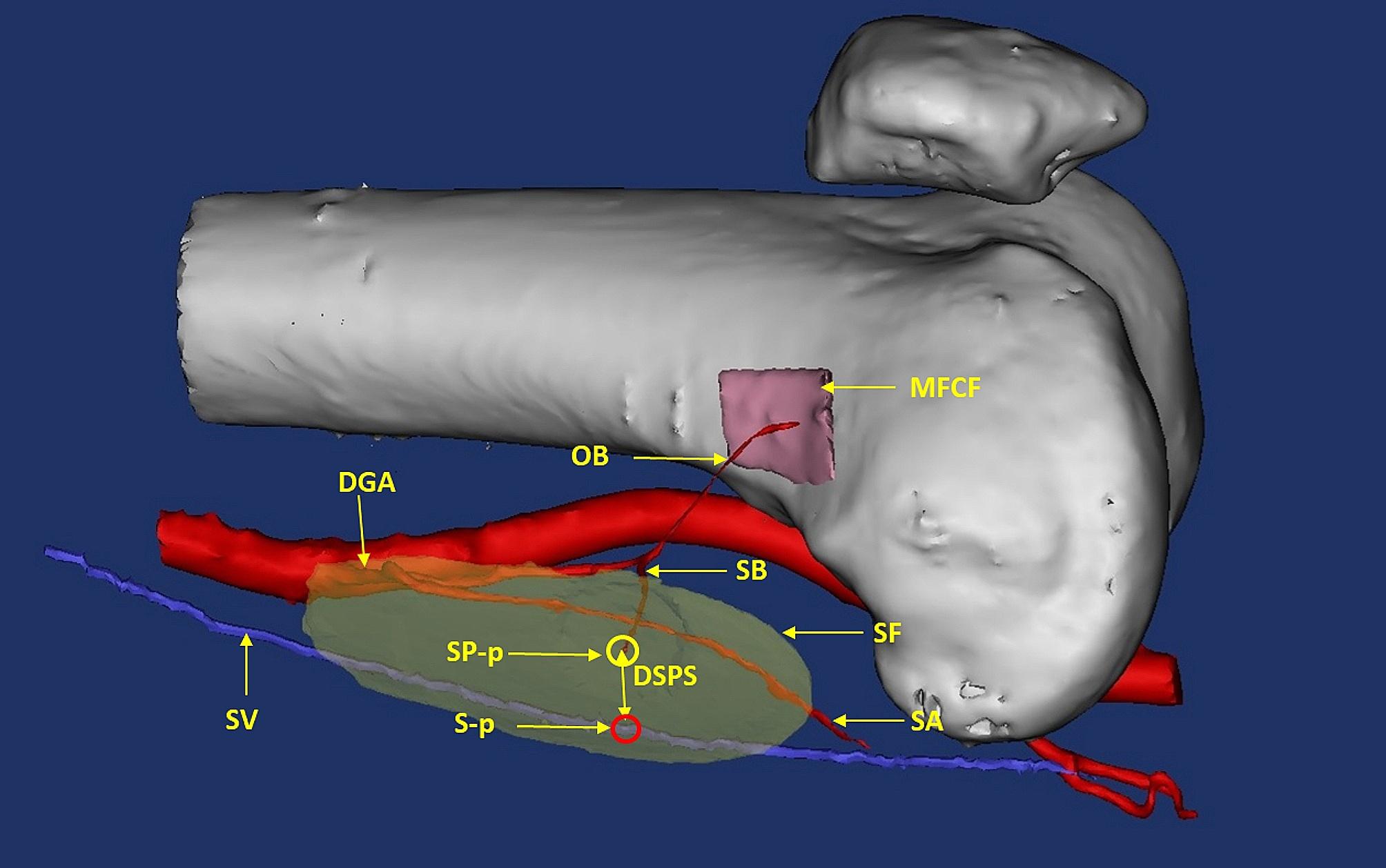
Digital design of a chimeric DGAP flap. In this case, the skin (SB) and bone (OB) perforators arose from the distal DGA, and the SA originated from the proximal DGA. SV, saphenous vein; DGA, descending genicular artery; MFCF, medial femoral condyle flap; OB, osteoarticular branch; SB, skin branch; SF, skin flap; SA, saphenous artery; DSPS, distance between SP-p and S-p

All patients underwent computed tomography angiography (CTA) assessment of the donor site. The CTA data were processed using Mimics 20.0 software to design the flap before surgery (Fig. 2). If any variations in vascular perforators were observed, such as the DGAP and OB not originating from the same trunk, the surgical plan was adjusted in advance. If necessary, a matching medial femoral condyle flap (MFCF) was designed for the bone defect in the recipient area. Postoperatively, CTA of the recipient area was performed to evaluate the blood supply to the MFCF. In patients with foot tissue defects, flaps were obtained from the contralateral medial femoral condyle area. The flap was designed using the medial longitudinal axis of the lower limb as the reference. To determine the location of the flap, Doppler ultrasound was used to detect the DGAP on the skin, which was found 4–6 cm proximal to the highest point of the medial femoral condyle. This location was marked as the P point, serving as the center point for the transverse diameter of the flap. The proximal end of the flap could be extended up to 20 cm from the P point, ensuring that the flap’s edge was positioned as far as possible from the knee joint.

### Surgical technique

**Fig. 3:**
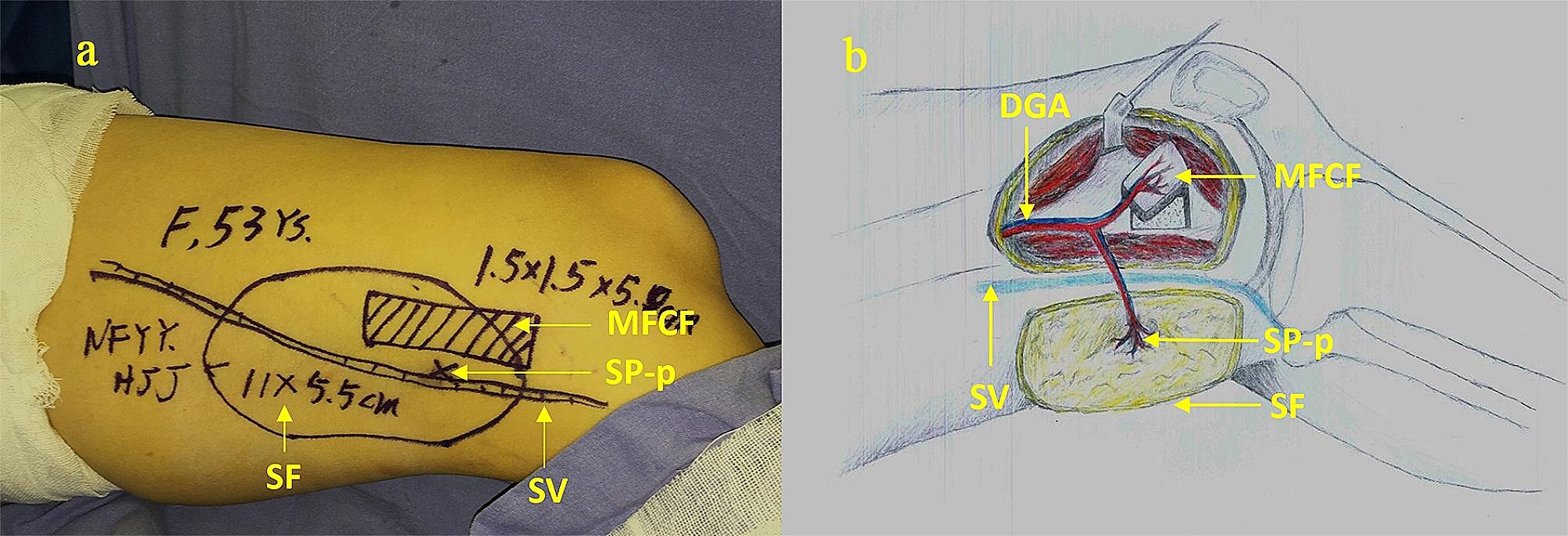
After the use of preoperative CTA and intraoperative Doppler ultrasound, the initial marking of the incision line in case 2 (**3a**). The entire chimeric DGAP flap (MFCF and SF) was completely isolated, and the SV was preserved (**3b**)

All patients were placed in a supine position and anesthetized with either general intubation or combined spinal and epidural anesthesia. The general profile of the flap and the SP-p were marked on the body surface (Fig. 3a). First, an anterior-medial thigh incision was made along the body surface marking line, and the SP-p of the DGAP was separated near the body surface marking point. Reverse separation along the SB to the origin of the DGA was performed. The muscular branches of the DGA were ligated, and the sartorius muscle was pulled to the medial side to find the adductor canal and the saphenous nerve, where the trunk of the DGA emerged from the adductor canal. During separation, the trunk of the great saphenous nerve along the DGA and the patellar anterior branch were observed. If a bone graft was needed, an adductor canal incision was made along the DGA to find the OB on the bone surface of the anterior-medial femoral condyle. This part of the periosteum and bone of the medial femoral condyle could be harvested as a bone-skin chimeric flap. The SV in this segment could be exposed with an intact membrane within the shallow deep fascia. The intersection point of the vertical line from the SP-p to the SV was marked as the S point (S-p). The DSPS of each patient was measured and recorded during surgery. The DGA was separated as far as possible and ligated at the origin of the superficial femoral artery. The flap was harvested with the SP-p as the midpoint to form an arteriovenous pedicle. A schematic diagram of flap harvesting is shown in Fig. 3b.

The free skin or chimeric flap was transferred to the recipient area with the radial artery or its branch as the supply artery for the upper limb, or the dorsal foot artery or anterior tibial artery as the supply artery for the lower extremity. Two accompanying veins were anastomosed in all flaps. All arteries and veins underwent end-to-end anastomosis.

## Results

The skin artery perforator was located in the medial femoral condyle plane, in front of the SV trunk, in all specimens. Both the skin artery perforator and the osteoarticular artery branches originated from the DGA. Except for one SA that originated later than the skin artery perforator, all others originated from the proximal DGA. The average distance between the DGAP and the SV was 3.56 ± 0.53 cm (range: 2.9–4.1 cm).

Patient demographics, injury cause, recipient site, and recipient vessel are summarized in Table 1. The average operative time was 3.2 h (range: 2.5–5.0 h), and the average hospitalization time was 18.6 days (range: 15–35 days). Intraoperatively, the mean pedicle length was 76.1 ± 17 mm. The average arterial diameter was 2.6 ± 0.5 mm, and the average venous diameter was 2.7 ± 0.7 mm. The mean flap area at the donor site was 5.2 × 10.3 cm. The average distance between the DGAP and the SV was 3.71 ± 0.38 cm (range: 2.9–4.3 cm). Table 2 presents the DGAP flap-related data. The average follow-up time was 11.23 months (range: 6–48 months). Thirty flaps completely survived, while one flap experienced partial necrosis but healed after two weeks of skin grafting. Six patients treated with skin–bone chimeric flaps showed healing on X-ray examination three months postoperatively. Five patients received free skin grafting in the donor area, while others underwent direct suture and healed with linear scars. Eight patients underwent skin flap thinning three to twelve months after surgery and experienced complication-free healing. All patients’ donor areas healed without numbness.

**Table 1:** Patient demographics, injury cause, recipient site, and recipient vessel

Characteristic	Recipient vessel	Result (%)
Sex		
Male		24 (77.4)
Female		7 (22.6)
Mean age ± SD, yrs.		30.9 ± 15.3
Smoking		6 (19.4)
Injury causes		
Machine injury		16 (51.6)
Traffic accident		13 (41.9)
Burn		2 (6.5)
Trauma types		
Skin defect of the foot	Arteriae dorsalis pedis	14 (45.2)
Skin defect of the hand	Radial artery or its branch	9 (29.0)
Thumb distal-segment defect	Radial artery	2 (6.5)
Bone–skin defect of the distal tibia	Arteriae tibialis anterior	2 (6.5)
Tendon exposure of the wrist	Radial artery	1 (3.2)
Bone–skin defect of the forearm	Arteria radialis	1 (3.2)
Bone–skin defect of the first metatarsal	Arteriae dorsalis pedis	1 (3.2)
Femoral head necrosis	Ascending branch of the lateral circumflex femoral artery	1 (3.2)

## Clinical cases

### Case 1

**Fig. 4:**
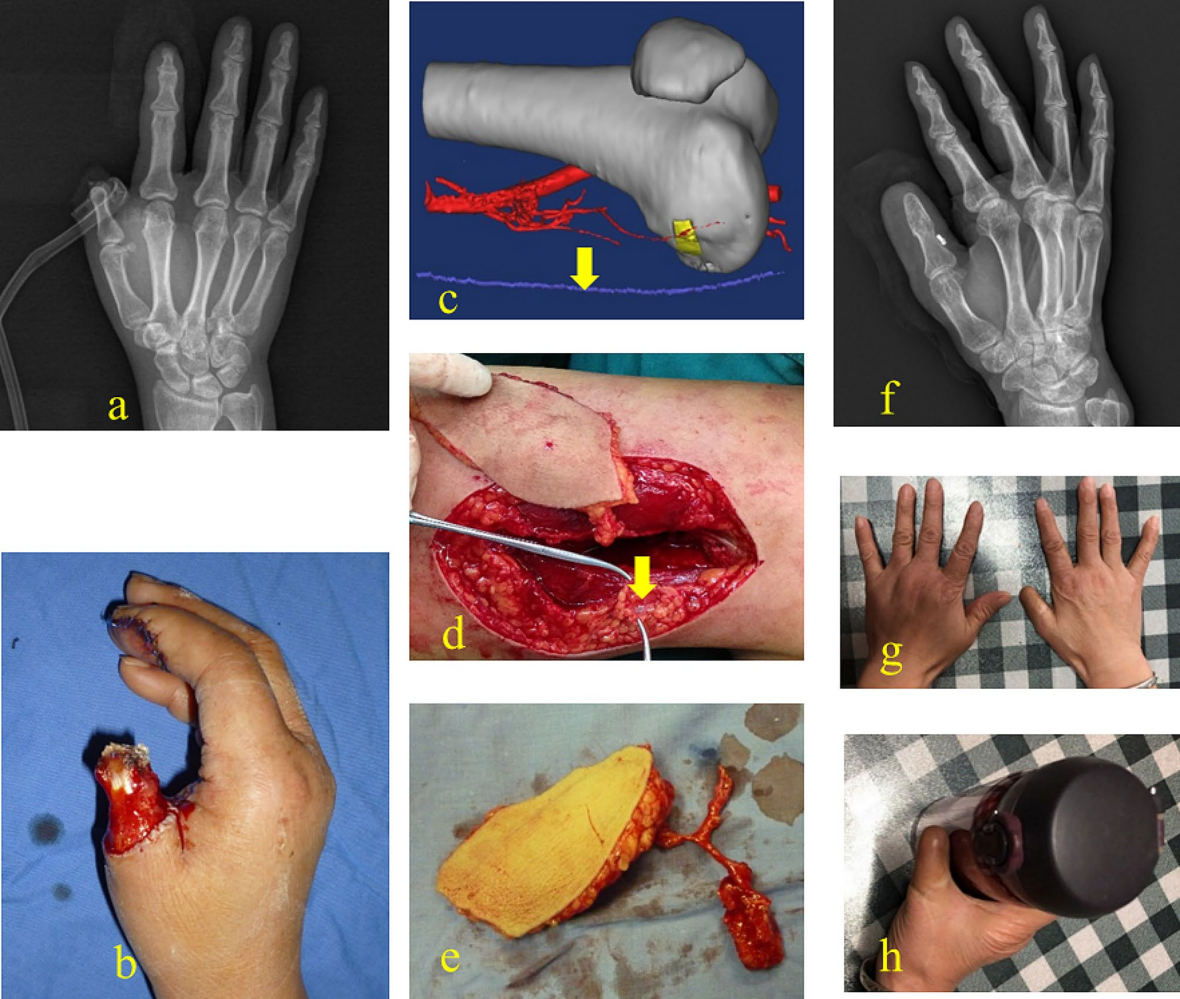
Case 1. Bone and skin defect of the right thumb before reconstruction (**a, b**). Preoperative plan of the chimeric DGAP flap using Mimics 20.0 with CTA data. The saphenous vein (yellow arrow) (**c**). Intraoperative photograph shows that the saphenous vein (yellow arrow) was preserved in the donor site (**d**), and the chimeric DGAP flap was harvested (**e**). Postoperative CTA of the recipient area, SB, skin artery branch, and OB (**f**). Radiograph of the same hand showing bony union 72 months after reconstruction and screw removal (**g**). Final outcome of the same thumb after reconstruction with a satisfactory appearance (**h**)

A 46-year-old female presented with right thumb III defects, soft-tissue defects in the proximal part of the thumb, and exposed phalangeal bone (Gustilo IIIb). The patient underwent emergency debridement and negative pressure drainage using a vacuum-assisted closure (VAC) device. Five days after injury, the terminal thumb was reconstructed, and the proximal phalangeal wound was covered with a DGA skin–bone chimeric flap without the SV. The DGA was anastomosed to the radial artery intraoperatively. The bone graft was fixed with a hollow screw and a Kirschner wire. Internal fixation was removed six months after surgery. The final appearance and function of the affected thumb and bony union are shown in Fig. 4.

**Table 2:** Descending genicular artery perforator flap-related data

Features	Result(%)
n	31(100)
Type of flap	
Skin flap	25(80.6)
Osteocutaneous chimeric flap	6(19.4)
Nerve	
Including medial femoral cutaneous nerve	10(32.3)
Excluding medial femoral cutaneous nerve	21(67.7)
Donor site	
Direct suture	26(83.9)
Skin grafting	5(16.1)
Pedicle length ± SD, mm	76.1 ± 17.0
Arterial diameter ± SD, mm	2.6 ± 0.5
Venous diameter ± SD, mm	2.7 ± 0.7
Flap size	
Width ± SD, cm	5.2 ± 1.7
Length ± SD, cm	10.3 ± 3.2
Distance between SP-p and S-p ± SD, cm	3.71 ± 0.38

### Case 2

**Fig. 5:**
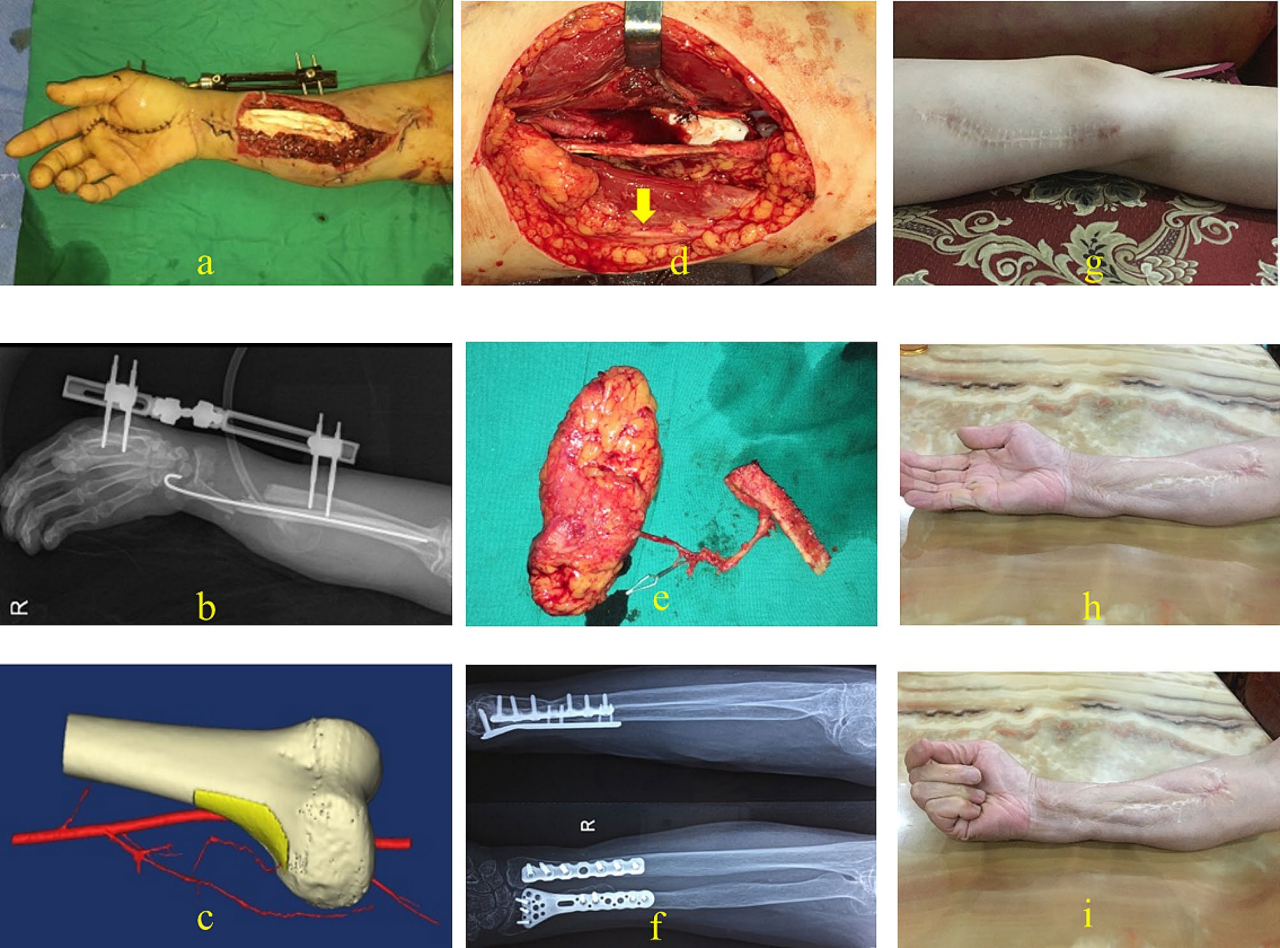
Case 2. Bone and soft-tissue defect of the right forearm after trauma (**a, b**). Preoperative plan of the DGAP flap without saphenous vein (**c**). Intraoperative picture shows that the saphenous vein (yellow arrow) was preserved (**d**). The harvested chimeric DGAP flap with the pedicle (**e**). After a follow-up period of 12 months, radiology showed a bony union (**f**). Final appearance of the donor site (**g**). Maintenance of finger motion and hemi- chimeric fist (**h, i**)

A 53-year-old female presented with an open fracture (Gustilo IIIb) on her right forearm caused by a machine injury. After emergency debridement and external fixation, soft-tissue and bone defects were covered using a VAC device for closed negative pressure drainage. A second debridement was performed, and the VAC device was replaced before the defect was repaired with a DGAP flap without the SV nine days after injury. The DGA was anastomosed to the radial artery intraoperatively. Ulnar and radial fractures were fixed using plates, and external fixation was retained. After three months, external fixation was removed, and flap thinning and tendon release were performed. The final function of the affected limb and bony union are shown in Fig. 5.

### Case 3

**Fig. 6:**
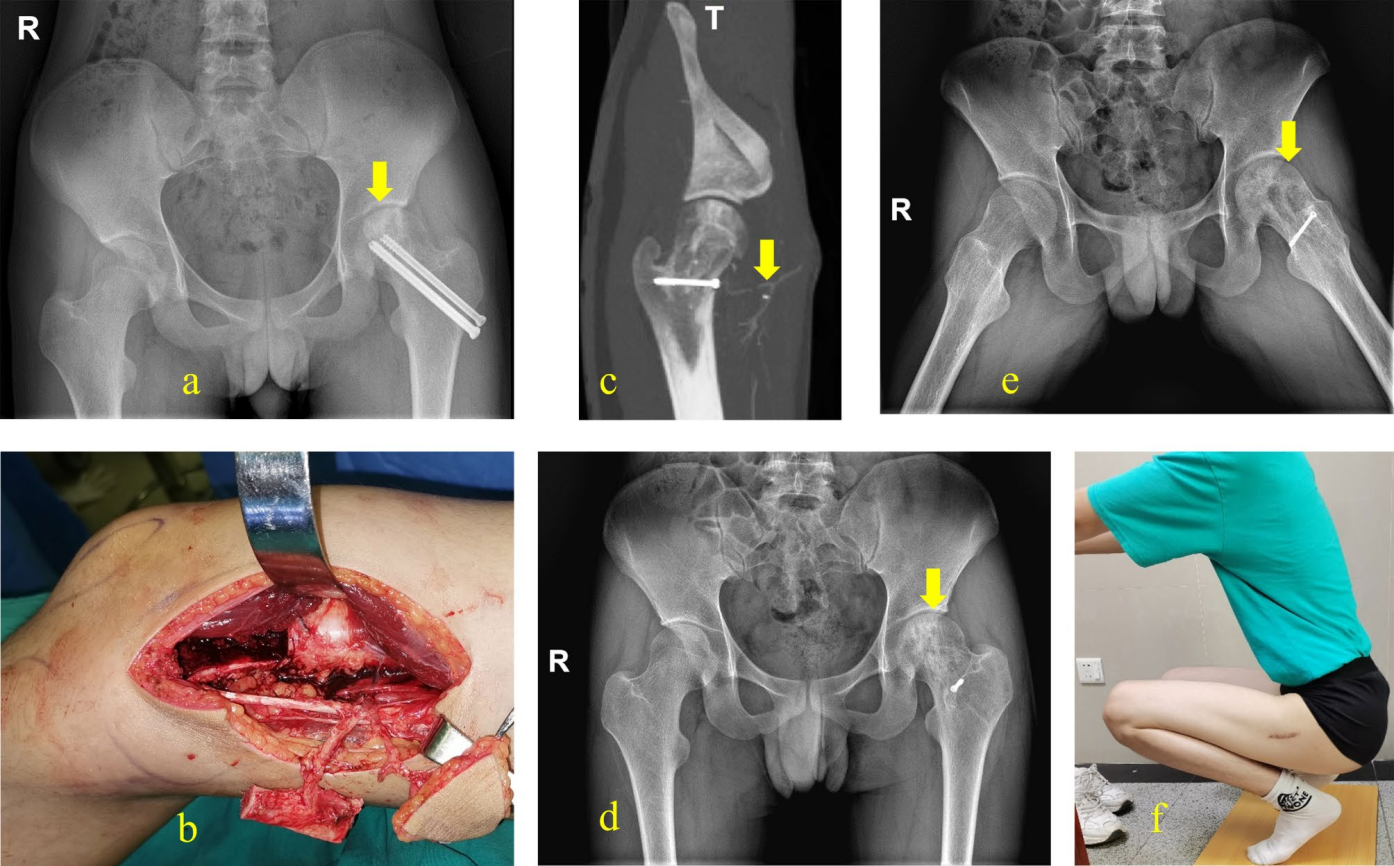
Case 3. Anteroposterior radiograph of the pelvis shows cystic degeneration (yellow arrow) in the femoral head(**a**).Intraoperative picture shows the harvested chimeric DGA flap with the pedicle(**b**). Postoperative CTA showed that the vascular anastomosis of the flap(yellow arrow) was unobstructed(**c**). After a follow-up of 48 months, there is no abnormality in the shape of the femoral head and the cystic degeneration has disappeared. (**d, e**). Final appearance of hip range of motion (**f**)

A 16-year-old patient presented with femoral head necrosis (ARCO stage III) one year after internal fixation for a femoral neck fracture. After completing CTA and other examinations, we proceeded with core decompression combined with chimeric DGAP flap transplantation. During the operation, the DGA was anastomosed to the ascending branch of the lateral circumflex femoral artery. Additionally, we implanted the removed femoral head bone into the defect at the flap donor site. Regular postoperative follow-up was conducted for four years to assess the function and images of the affected limb, as shown in Fig. 6 and Supplemental Digital Content 1.

## Discussion

In this study, no communicating branch was found between the SV and the descending genicular vein, indicating that the flap does not require the SV trunk. The average distance between the descending genicular artery perforator and the SV was 3.71 ± 0.38 cm (range: 2.9–4.3 cm). All flaps of the 31 patients survived without any venous crisis, suggesting that the DGAP flap can survive solely by utilizing the descending genicular artery (DGA) and vein, without the SV. This study demonstrated positive outcomes in treating avascular necrosis of the femoral head using the chimeric DGAP flap. The findings suggest that the chimeric flap holds promise as a potential donor for bone graft treatment in cases of femoral head necrosis.

A digital technique was used preoperatively to design an SA flap for six patients, but the range of the flap incision affected knee joint flexion and extension. Previously, some authors [16] attempted to use bone wax to simulate the size and shape of the skull defect area for effective use of the DGA bone flap, but this was not accurate enough. Recently, some experts [17] have employed 3D printing technology to create a model similar in shape to the bone defect area. While this helped in accurately harvesting the free MFCF during surgery, it did not assist in separating the most critical vascular perforator. In this study, we used Mimics 20.0 software to preoperatively simulate the shape of the flap or chimeric flap. We analyzed the vascular variation and anatomical relationship, providing guidance for flap harvesting. The largest successfully harvested free bone flap had an area of 5.0 × 1.5 × 1.5 cm (Case 2).

An early report [2] of the free SA flap suggested that it was necessary to cut the sartorius muscle to protect the artery perforator. However, DGAP flap harvesting without the SV can preserve the saphenous nerve, the SV, and the adjacent muscles of the knee joint. The skin branch is a direct cutaneous perforator that can be separated using a tourniquet. During surgery, the main trunk of the SV and other adjacent vessels can be easily protected. Cutaneous, muscular, and osteoarticular DGAPs can be used to form the chimeric tissue flap for repairing open bone defects, which can control local infection. This is recommended as the preferred free bone graft donor area for small bone defects [6, 18–20]. Satisfactory results were obtained using DGAP osteocutaneous flaps to treat 6-cm radial bone defects and thumb III defects. While some authors have used the lateral upper arm flap with a humeral external condyle bone graft to repair thumb defects [21], the lateral upper arm osteocutaneous flap is smaller than the DGAP osteocutaneous flap, and the DGAP donor area is more concealed. The free DGAP bone flap has also been used to repair thumb osteomyelitis and nonunion [8, 19]. In this study, the soft-tissue defect was treated with full-thickness skin grafting on the bone flap, and the chimeric flap was used to repair the composite tissue defect.

Necrosis of the femoral head is a long-term complication of femoral neck fracture [22]. Currently, the main methods for vascularized bone graft include the use of a free vascularized fibula, free vascularized iliac bone, or a pedicled iliac bone flap [23, 24]. However, the fibula graft carries the risk of sural nerve injury and secondary ankle deformity [25], while the iliac graft has the potential for anterolateral femoral cutaneous nerve injury and subcutaneous hematoma [26]. In this study, we employed the descending genicular artery perforator chimeric flap without the saphenous vein to treat a patient with ARCO stage III femoral head necrosis (Case 3). The patient underwent regular follow-up for four years post-surgery, and it was observed that the progression of avascular necrosis of the femoral head had halted, with an excellent Harris score. The advantage of this osteocutaneous flap is that it causes less damage to the donor site. Moreover, when using the flap for bone grafting, it can act as a monitor to reflect the blood supply of the transplanted bone within the femoral head. There has been no similar report in the previous literature on DGA flaps [18, 27].

Multiple studies have indicated that MCFC flaps are associated with low donor site morbidity rates, although complications like chronic knee pain and paresthesia can still arise [28, 29]. In our research, efforts were made to preserve the main trunk of the SV, the saphenous nerve, and the muscles surrounding the knee joint as much as possible during DGAP flap harvesting. This approach was designed to minimize any negative effects on the function and appearance of the knee in the donor region. During the follow-up period, temporary swelling was observed and attributed to excessive suture tension. Therefore, based on our cadaveric anatomical studies and clinical statistical results, we suggest it is necessary to limit the flap width to within 7 cm and to consider a BMI ≥ 28 as a contraindication for this flap. Patients in the study did not report any long-term discomfort, such as knee pain or skin numbness, after the repair. However, the thickening of subcutaneous fat at the donor site presented a challenge in distinguishing between SB and flap thinning. Although no complications were observed in this study, previous research noted femoral fractures in the donor region after harvesting DGAP bone flaps [30, 31]. We consequently recommend considering allograft or artificial bone transplantation in the donor region and promoting the use of crutches or a walker to bear weight for 2–3 months post-surgery.

This research has two limitations. Firstly, it is a retrospective study with a small number of patients. Although our case series is limited by a small sample size, it highlights the potential utility of the DGAP chimeric flap without the saphenous vein for locoregional reconstruction. Secondly, the surgery has a long learning curve due to the various anatomical variations in the branches of the descending genicular artery.

## Conclusion

The main trunk of the SV can be preserved during DGAP flap harvesting, causing less damage to the donor site and having no effect on flap survival. Attention should be paid to protecting the SV when the distance between the posterior edge of the flap and the skin branch exceeds 3.7 cm. DGAP flaps without the SV are suitable for repairing skin-bone composite tissue defects, such as thumb III defects. This chimeric flap is expected to be a potential donor for the treatment of avascular necrosis of the femoral head.

### Electronic supplementary material

Below is the link to the electronic supplementary material.

Supplementary Material 1

## Data Availability

To protect patient privacy, the datasets used and/or analyzed during the current study are available from the corresponding author on reasonable request.

## Abbreviations

DGA: Descending Genicular Artery
DGAP: Descending Genicular Artery Perforator
SV: Saphenous Vein
SA: Saphenous Artery
VAC: Vacuum-Assisted Closure
OB: Osteoarticular Branch
SB: Skin Branch
CTA: Computed Tomography Angiography

## References

[1] Giesen T, Costa F, Fritsche E. Complex reconstruction of the clavicle with a prefabricated medial femur condyle chimeric flap including a superficial circumflex iliac artery perforator flap: a case report. Microsurg; 2023. 10.1002/micr.31108 37668043

[2] Acland RD, Schusterman M, Godina M (1981). The saphenous neurovascular free flap. Plast Reconstr Surg.

[3] Scaglioni MF, Rodi T, Fritsche E (2021). The versatility of the pedicled medial sural artery perforator flap: from simple to its chimeric pattern and clinical experience with 37 cases. Plast Reconstr Surg.

[4] Zhang YZ, Wen SZ, Zhang HQ (2016). Three-dimensional digitalized virtual planning for saphenous artery flap: a pilot study. Comput Assist Surg (Abingdon England).

[5] Karam Rsel S, Elebio Lu S (2006). Use of the medial side of the knee skin as a free flap: saphenous flap. Plast Reconstr Surg.

[6] Sananpanich K, Kraisarin J (2015). Descending genicular artery free flaps: multi-purpose tissue transfers in limb reconstruction. J Plast Reconstr Aesthetic Surg.

[7] Mattos D, Ko JH, Iorio ML (2019). Wrist arthrodesis with the medial femoral condyle flap: outcomes of vascularized bone grafting for osteomyelitis. Microsurg.

[8] Rossello C, Antonini A, Zoccolan A, Burastero G, Rossello MI (2019). Reconstructive surgery for thumb osteomyelitis: a new way of remodelling the vascularized medial femoral condyle flap. A case report. Handchir Mikrochir Plast Chir.

[9] Nakanishi A, Omokawa S, Kawamura K, Shimizu T, Tanaka Y (2019). Vascularized medial femoral condyle graft for nonunion after failed radiolunate arthrodesis. Case Rep Plast Surg Hand Surg.

[10] Caggiati A, Bergan JJ (2002). The saphenous vein: derivation of its name and its relevant anatomy. J Vasc Surg.

[11] Fonkoue L, Behets C, Steyaert A (2021). Anatomical study of the descending genicular artery and implications forimage-guided interventions for knee pain. Clin Anat.

[12] Mendoza E (2021). Anatomie Der Vsaphena magna und parva. Phlebologie.

[13] Xu Q, Zheng X, Li Y, Zhu L, Ding Z (2020). Anatomical study of the descending genicular artery chimeric flaps. J Invest Surg.

[14] Hirtler L, Lubbers A, Rath C (2019). Vascular coverage of the anterior knee region - an anatomical study. J Anat.

[15] Cang Z, Xu Y, Wang M, Xu M, Yuan S (2021). Anterior tibial artery injury is not the contraindication of medial plantar flap: digital subtraction angiography evidence and clinical application. J Plast Reconstr Aesthetic Surg.

[16] Kazmers NH, Rozell JC, Rumball KM et al. Medial femoral condyle microvascular bone transfer as a treatment for capitate avascular necrosis: surgical technique and case report. J Hand Surg-Am 2017;42. 10.1016/j.jhsa.2017.04.006 28495027

[17] Schmidt M, Holzbauer M, Kwasny O, Huemer GM, Froschauer S (2020). 3d printing for scaphoid-reconstruction with medial femoral condyle flap. Injury.

[18] Kazmers NH, Thibaudeau S, Steinberger Z, Scott LL (2018). Upper and lower extremity reconstructive applications utilizing free flaps from the medial genicular arterial system: a systematic review. Microsurg.

[19] Rodriguez JR, Chan JK, Huang R (2022). Free medial femoral condyle flap for phalangeal and metacarpal bone reconstruction. J Plast Reconstr Aesthetic Surg.

[20] Kazmers NH, Thibaudeau S, Gerety P, Lambi AG, Levin LS (2018). Versatility of the medial femoral condyle flap for extremity reconstruction and identification of risk factors for nonunion, delayed time to union, and complications. Ann Plas Surg.

[21] di Summa PG, Higgins G, Cotrufo S (2022). Distal brachial artery perforator flap: a new chimeric option for complex hand and digits defects. J Plast Reconstr Aesthetic Surg.

[22] Kim C, Shin M, Lee D, Choi SJ, Moon DH (2022). Hidden osteonecrosis of the femoral head after healed femoral neck fractures: magnetic resonance imaging study of 58 consecutive patients. Arch Orthop Traum Su.

[23] Wan J, Hu Y, Li J, Zeng Y, Ren H (2022). Comparison of the outcome of different bone grafts combined with modified core decompression for the treatment of arco ii stage femoral head necrosis. Int Orthop.

[24] Hu L, Deng X, Wei B, Wang J, Hou D. Comparative analysis of surgical interventions for osteonecrosis of the femoral head: a network meta-analysis of randomized controlled trials. J Orthop Surg Res 2023;18. 10.1186/s13018-023-04463-4 PMC10722734 38098128

[25] Chen W, Du W, Wu P (2023). Outcomes of free vascularized iliac bone flap for severe traumatic osteonecrosis of femoral head in young adults. Eur J Trauma Emerg S.

[26] Lei P, Du W, Liu H et al. Free vascularized iliac bone flap based on deep circumflex iliac vessels graft for the treatment of osteonecrosis of femoral head. J Orthop Surg Res 2019;14. 10.1186/s13018-019-1440-2 PMC6883673 31779640

[27] Mohan AT, Zhu L, Morsy M (2019). Reappraisal of perforasomes of the superficial femoral, descending genicular, and saphenous arteries and clinical applications to locoregional reconstruction. Plast Reconstr Surg.

[28] Politikou O, Wirth S, Giesen T (2020). Corticoperiosteal medial femoral condyle flap for recalcitrant nonunion in ankle and foot: outcomes and radiological evaluation of donor site morbidity. Foot Ankle Surg.

[29] Mehio G, Morsy M, Cayci C (2018). Donor-site morbidity and functional status following medial femoral condyle flap harvest. Plast Reconstr Surg.

[30] Haines M, Baba M, Stewart DA. Iatrogenic femur fracture following medial femoral condyle flap harvest. J Hand Surg-Am 2020;45. 10.1016/j.jhsa.2019.12.001 32089380

[31] Son JH, Giladi AM, Higgins JP (2019). Iatrogenic femur fracture following medial femoral condyle flap harvest eventually requiring total knee arthroplasty in one patient. J Hand Surg-Eur Vol.

